# Navigating the Uncertainty of B3 Breast Lesions: Diagnostic Challenges and Evolving Management Strategies

**DOI:** 10.3390/jpm15010036

**Published:** 2025-01-18

**Authors:** Sabatino D’Archi, Beatrice Carnassale, Alejandro Martin Sanchez, Cristina Accetta, Paolo Belli, Flavia De Lauretis, Enrico Di Guglielmo, Alba Di Leone, Antonio Franco, Stefano Magno, Francesca Moschella, Maria Natale, Lorenzo Scardina, Marta Silenzi, Riccardo Masetti, Gianluca Franceschini

**Affiliations:** 1 Multidisciplinary Breast Centre, Department of Women’s and Children’s Health Sciences and Public Health, Fondazione Policlinico Universitario A. Gemelli IRCCS, 00168 Rome, Italy; 2 Department of Diagnostic Imaging, Oncological Radiotherapy and Hematology, Fondazione Policlinico Universitario Agostino Gemelli IRCCS, 00168 Rome, Italy; 3 Department of Medical and Surgical Sciences, Catholic University of the Sacred Heart, 00168 Rome, Italy

**Keywords:** B3 breast lesions, diagnosis, imaging techniques, biopsy methods, breast surgery, AI in breast tumor screening, genomic analysis in breast tumor diagnosis

## Abstract

B3 breast lesions, classified as lesions of uncertain malignant potential, present a significant diagnostic and therapeutic challenge due to their heterogeneous nature and variable risk of progression to malignancy. These lesions, which include atypical ductal hyperplasia (ADH), papillary lesions (PLs), flat epithelial atypia (FEA), radial scars (RSs), lobular neoplasia (LN), and phyllodes tumors (PTs), occupy a “grey zone” between benign and malignant pathologies, making their management complex and often controversial. This article explores the diagnostic difficulties associated with B3 lesions, focusing on the limitations of current imaging techniques, including mammography, ultrasound, and magnetic resonance imaging (MRI), as well as the challenges in histopathological interpretation. Core needle biopsy (CNB) and vacuum-assisted biopsy (VAB) are widely used for diagnosis, but both methods have inherent limitations, including sampling errors and the inability to determine malignancy in some cases definitively. The therapeutic approach to B3 lesions is nuanced, with treatment decisions strongly influenced by factors such as the lesion size, radiological findings, histopathological characteristics, and patient factors. While some lesions can be safely monitored with watchful waiting, others may require vacuum-assisted excision (VAE) or surgical excision to rule out malignancy. The decision-making process is further complicated by the discordance between the BI-RADS score and biopsy results, as well as the presence of additional risk factors, such as microcalcifications. This review provides an in-depth analysis of the current diagnostic challenges and treatment strategies for B3 lesions, emphasizing the importance of a multidisciplinary approach to management. By synthesizing the most recent research, this article aims to provide clinicians with a clearer understanding of the complexities involved in diagnosing and treating B3 breast lesions while highlighting areas for future research, such as artificial intelligence and genomics, to improve the diagnostic accuracy and patient outcomes.

## 1. Introduction

Breast cancer is the most frequently diagnosed cancer and the second leading cause of cancer-related mortality in women [1]. The treatment of breast cancer involves a comprehensive, multidisciplinary strategy, including surgical oncology, radiotherapy, and medical oncology, which, together with the implementation of screening programs, have been correlated with a decline in breast cancer-related deaths. Radiological screening for breast cancer often leads to a biopsy to obtain a diagnosis. Imaging assessments, typically mammography, breast ultrasound, or both, are employed to identify lesions requiring a biopsy and plan the biopsy technique and procedure. Abnormalities detected on imaging are classified based on their probability of malignancy according to the Breast Imaging-Reporting and Data System (BI-RADS) [2]. A histological diagnosis can be achieved through either CNB or VAB. Biopsy findings can be categorized into five categories: B1 (normal breast tissue or inadequate sample), B2 (benign lesion), B3 (uncertain malignant potential), B4 (high suspicion of malignancy), and B5 (malignant lesion) [3,4].

The B3 category has an incidence that ranges from 3% to 21%, with higher rates found in the screening-age population [5,6]. It includes several conditions: atypical ductal hyperplasia (ADH), flat epithelial atypia (FEA), classical lobular neoplasia (LN), papillary lesions (PLs), radial scars (RSs), and benign or borderline phyllodes tumors (PTs). Additionally, it encompasses various miscellaneous entities such as fibroepithelial lesions (FELs), mucocele-like lesions, and apocrine adenosis.

Because they occupy a “grey zone” between benign and malignant conditions, their diagnosis and management can be complex and often controversial. The diagnostic evaluation of B3 lesions is particularly challenging. Imaging techniques such as mammography, ultrasound, and MRI provide valuable information but are often insufficient to distinguish benign from malignant lesions. Biopsy, including CNB and VAB, is essential but has limitations, such as sampling errors and difficulty accurately assessing the malignant potential. Discrepancies between biopsy results and imaging findings further complicate decision-making, as apparently benign lesions may exhibit malignant features and vice versa. Furthermore, B3 lesions are often associated with risk factors such as microcalcifications, which complicate imaging interpretation and influence management decisions. This uncertainty, combined with the diverse nature of B3 lesions, highlights the importance of a multidisciplinary approach involving radiologists, pathologists, and surgeons for accurate diagnosis and optimal treatment planning.

Management strategies for B3 lesions are individualized, considering the lesion size, radiological findings, histopathological features, and patient risk factors. Some lesions can be safely monitored with watchful waiting, while others may require more invasive interventions such as VAE or surgical excision to rule out malignancy.

In addition to these ongoing challenges, new technologies, such as artificial intelligence (AI), are emerging in breast cancer research. AI shows promise in improving diagnostic accuracy and imaging interpretation. When combined with molecular and genetic profiling, AI could provide proper insights into the malignant potential of B3 lesions, enabling more precise and personalized treatment strategies.

This study aims to summarize, category by category, the various recommendations related to surgical treatment, radiological management, and the upgrade rate specific to each histological subtype, highlighting the need for a multidisciplinary approach and the potential of new tools to improve diagnostic accuracy and patient outcomes.

## 2. Existing Evidence Regarding Diagnostic and Therapeutic Approach

### 2.1. Atypical Ductal Hyperplasia (ADH)

#### 2.1.1. Radiology and Pathology

**Figure 1:**
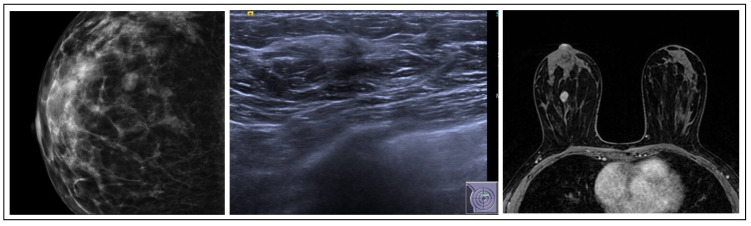
ADH radiological features.

ADH is one of the most frequently recognized B3 lesions in breast pathology. It often correlates with grouped microcalcifications, nodules, or atypical densities visible on mammograms. Predominantly, ADH is detected in cases where calcifications appear on mammograms, constituting a significant majority (81.6%), while, in other studies, the prevalence of ADH detected alongside microcalcifications is even higher (86%) [7]. The MRI in these cases shows a lesion with nonspecific characteristics, such as a focal non-mass area or a small, irregular mass (Figure 1). Histologically, ADH is a small, low-grade, clonal intraductal lesion, that generally measures up to 2 mm in maximum diameter or consists of segments of a terminal ductal lobular unit.

#### 2.1.2. Upgrade Rate

Differentiating atypical ductal hyperplasia (ADH) from low-grade ductal carcinoma in situ (DCIS) is based solely on the size of the lesion. Lesions larger than 2 mm are classified as low-grade DCIS. Because this classification cannot be reliably determined through preoperative core needle biopsy (CNB) or vacuum-assisted biopsy (VAB), additional sampling of the lesion is required through vacuum-assisted excision (VAE) or surgical removal [8].

Consistently, the published literature underscores that the likelihood of ADH being upgrading to malignancy is higher with smaller samples, such as those obtained using 14 G cores, compared to VAB specimens. The upgrade rate for ADH ranges from 18% to 87% for 14 G needles, contrasting with 10% to 39% for 11 or 9 G samples, with a combined positive predictive value of 21% from vacuum-assisted sampling. Fundamentally, it is logical that using a bigger tissue sample lowers the chances of overlooking a diagnosis of DCIS or invasive cancer [9].

Numerous studies have documented an upgrade rate of ADH to malignancy ranging from 5% to 50%. Elements linked to a heightened likelihood of this upgrade consist of acquiring a smaller quantity of biopsy material, primarily via CNB; a lack of correlation between calcifications in ADH and imaging findings; the persistence of microcalcifications following VAB; lesion dimensions surpassing 15 mm on imaging; the patient age exceeding 50 years; and the presence of multifocality in the ADH biopsy sample [8,10].

#### 2.1.3. Current Indications

The recently published guidelines from the 3rd International Consensus Conference on B3 lesions recommend open surgical excision following a diagnosis of ADH obtained through CNB. This approach is also suggested as the preferred action if ADH is diagnosed via vacuum-assisted biopsy (VAB). However, for small or focal ADH lesions observed on imaging, a second vacuum-assisted excision (VAE) procedure may be considered after thorough discussion within a multidisciplinary team [11].

According to the guidelines from the UK National Health Service Breast Screening Programme (NHS BSP) Working Group, secondary VAE is recommended for further detailed assessment of most B3 lesions, regardless of whether they were initially diagnosed via CNB or primary diagnostic VAB [6].

European guidelines established by EUSOMA, EUSOBI, ESSO, and ESP emphasize the importance of surgical excision for atypical ductal hyperplasia (ADH) diagnosed through core needle biopsy (CNB) or vacuum-assisted biopsy (VAB), especially if the lesion is visible on imaging studies. For lesions smaller than 15 mm, image-guided vacuum-assisted excision (VAE) may be considered as an option. However, if the lesion diagnosed by CNB or VAB is larger than 15 mm, surgical excision is recommended, although VAE may still be an option worth considering.

### 2.2. Papillary Lesions (PLs)

#### 2.2.1. Radiology and Pathology

PLs typically appear in radiology as clearly outlined nodules, which may or may not have cystic characteristics, or as small masses within a dilated duct, sometimes showing vascularization on ultrasound. Mammography may depict normal findings or reveal clustered calcifications in 25% of cases. Single lesions are frequently located in the retroareolar region, whereas multiple lesions are commonly found in the periphery [12].

**Figure 2:**
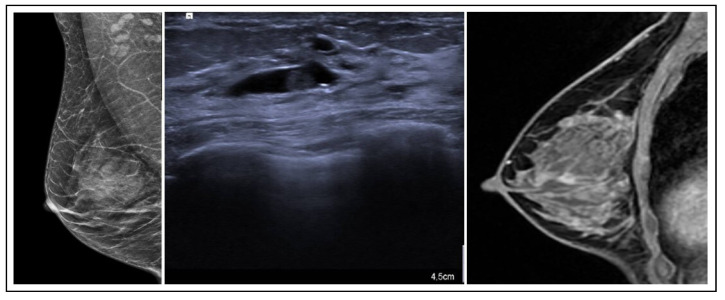
PL radiological features.

On MRI, PLs typically manifest as circumscribed, solid enhancing lesions; however, they may also exhibit irregular shapes and indistinct margins [13] (Figure 2). These lesions have a benign papillary structure with fibrovascular cores and benign epithelium, often classified as B3 due to potential variations.

#### 2.2.2. Upgrade Rate

The existence of epithelial atypia serves as a crucial indicator for potential malignancy upgrades and must be thoroughly recorded. When a lesion is devoid of epithelial atypia, the likelihood of malignancy in the resulting excision specimen is low, ranging from 9% to 13.2%. Conversely, when atypia is identified, the upgrade rate considerably increases, falling between 36% and 47.8% [14]. Recent research has indicated a median upgrade rate for lesions without atypia to ductal carcinoma in situ (DCIS) or invasive carcinoma (IC) of merely 2.3%; however, lesions with atypia exhibited a significantly elevated upgrade rate to DCIS or IC, with a median of 26.9% [15,16]. A recent meta-analysis conducted by Zhang et al. [17] highlighted ten upgrade predictors, including BI-RADS 4C or 5 classifications, palpable lumps, bloody discharge from the nipple, the presence of a mass or calcifications on mammography, radio-pathological discrepancies, peripheral lesion location, and sizes exceeding 1 cm. The upgrade rates associated with these factors varied from 7.3% to 31.1%.

#### 2.2.3. Current Indications

Retrospective studies suggest that if CNB yields a PL without atypia, VAB is a viable strategy. Surgical excision is necessary if CNB or VAB findings indicate a papilloma with atypia [18]. European guidelines [10] advocate surgical excision for PLs with atypia, while those without atypia can be safely managed with VAB and, if entirely excised, imaging surveillance. The 3rd Consensus Conference [11] focuses solely on pure PLs without atypia and equally recommends surgical excision or therapeutic VAE after a CNB-based PL diagnosis.

### 2.3. Radial Scars

#### 2.3.1. Radiology and Pathology

**Figure 3:**
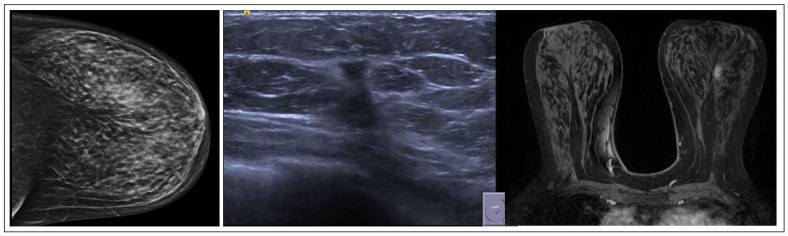
RS radiological features.

RSs usually appear on mammography as a spiculate lesion or a region of structural distortion with a radiolucent core, sometimes accompanied by calcifications. Tomosynthesis aids in identifying RSs on mammograms [19]. The appearance can vary on ultrasound, ranging from no distinct correlation to a hypoechoic irregular mass. Moreover, MRI findings may exhibit variability and lack specificity. RSs may present without any MRI correlation, displaying no enhancement, or manifest as mass lesions with irregular margins or non-mass enhancement [20]. These imaging characteristics require caution, as RSs can resemble invasive breast cancer (Figure 3). Histologically, central fibroelastosis is surrounded by compressed glandular structures and cysts, sometimes associated with benign or malignant changes [21].

#### 2.3.2. Upgrade Rate

The upgrade rate of RSs is significantly influenced by the existence of simultaneous atypical epithelial growth. Data reported on the upgrade rate from RSs to DCIS or invasive carcinoma show considerable variation, with estimates falling between 0% and 40% [22]. The quantity and size of the biopsy samples also influence the upgrade rate. For instance, Farshid et al. [23], in a systematic review and meta-analysis, demonstrated higher upgrade rates with CNB (5%) compared to VAB (1%). Numerous studies have shown that RS upgrade rates increase in the presence of atypia in the biopsy specimen. Racka et al. [21] observed a 9% malignant progression rate after the surgical removal of radiologically benign lesions without atypia on core needle biopsy, compared to a 36% rate for those with atypia. The NHSBSP Guidelines report upgrade rates of 36% for atypical cases versus 10% for non-atypical ones [6]. Other studies found upgrade rates of 28% for atypia and 4% without [24]. Additionally, Quinn et al. surveyed a large cohort, reporting 9% upgrade rates for lesions without atypia and 33% for those with atypia [25].

#### 2.3.3. Current Indications

European guidelines [10] advocate for VAE in RSs without atypia identified through CNB and surgical excision in RSs presenting with atypia, although VAE could be contemplated. The 3rd International Consensus Conference indicated that after RS detection without atypia on CNB, along with the imaging size, 58% of the panel endorsed therapeutic VAE. When the target lesion was completely excised, the majority of the panel (82%) preferred radiological follow-up after diagnostic VAB or VAE [15]. The AGO advises against operative excision for lesions under 5 mm or those nearly completely removed by VAB. Further re-excision is unnecessary if the lesion borders the resection margin [26]. The NHSBSP suggests comprehensive sampling with VAE, and depending on the outcomes, RSs without atypia necessitate no further monitoring, with patients reverting to routine mammographic screening (follow-up every 3 years). For RSs with atypia, consideration should be given to either open excision or annual mammographic follow-up, based on the decision of the multidisciplinary team [6].

### 2.4. Flat Epithelial Atypia (FEA)

#### 2.4.1. Radiology and Pathology

**Figure 4:**
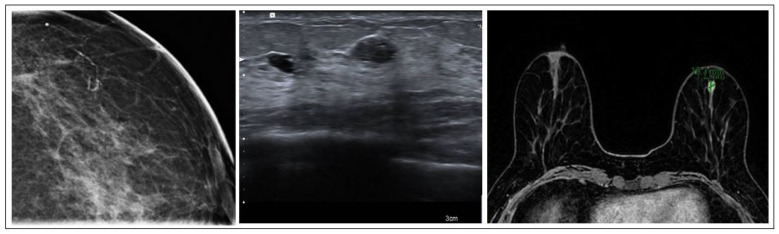
FEA radiological features.

FEA is commonly found alongside other suspicious lesions and exhibits imaging characteristics similar to both malignant and benign lesions. When observed on mammography, it manifests as clustered calcifications, while on ultrasound, it may manifest as an irregular hypoechoic mass. In MRI scans, FEA might be concealed or manifest as either a mass or a non-mass area exhibiting nonspecific characteristics [27] (Figure 4). FEA is histologically categorized within the spectrum of columnar cell lesions in the breast. This spectrum includes columnar cell alteration and hyperplasia, both of which lack atypia; however, the presence of atypia is what classifies it specifically as FEA [28].

#### 2.4.2. Upgrade Rate

The upgrade rate for FEA is uncertain and is often linked to the presence of concurrent lesions [27]. While the risk of progression to carcinoma for isolated FEA is minimal, Wahab et al. [29] found that the upgrade rate for pure FEA on core needle biopsy (CNB) after surgical excision is 5%. In contrast, Verschuur-Maes et al. [30] reported a 17% upgrade rate. Other recent studies indicate upgrade rates between 1% and 16% [26,31]. Like other B3 lesions, FEA requires further sampling since the presence of coexisting proliferative lesions increases the upgrade rate. Recent data, including individual trials, advocate therapeutic VAE with radiological monitoring if more than 90% of calcifications have been excised [29,32]. Unfortunately, these studies only analyze cases of microcalcifications and disregard other radiological findings.

#### 2.4.3. Current Indications

Several international guidelines recommend a single case discussion or radiological monitoring as the preferred approach following the diagnosis of FEA via VAB. Only situations demonstrating mass lesions, pathological–radiological incongruity or cases with many residual calcifications post-biopsy should undergo surgical excision [33,34]. Age, imaging, additional BC risk factors, lesion size, and correlations with calcification, in addition to the radiological–pathological alignment of FEA, constitute critical elements for informed decision-making [35]. In line with the WHO Working Group, observation could be deemed an acceptable management strategy in cases of pure FEA if radiological–pathological correlation is assessed [28]. The 3rd International Consensus Conference [11] suggests VAE or surgical excision upon FEA detection on CNB and surveillance if over 90% of the target lesion is removed on VAB. According to AGO guidelines, surgery may be avoided in instances of small lesions or nearly complete (>90%) eradication of calcifications [33]. NHS BSP recommends surgery solely in cases of radio-pathological discordance [6]. European guidelines [10] advocate surveillance in cases of pure FEA diagnosed on CNB or VAB concordant with imaging, VAE or surgical excision in instances of FEA with ADH diagnosed on CNB, VAE or surgical excision in cases of FEA with ADH diagnosed on VAB if not all calcifications are removed, or in cases of pathological–radiological incongruity.

### 2.5. Lobular Neoplasia (LN)

#### 2.5.1. Radiology and Pathology

**Figure 5:**
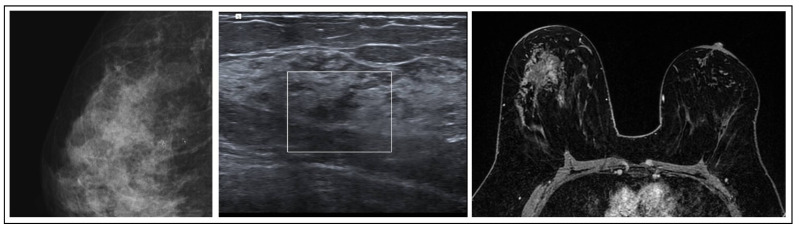
LN radiological features.

LN is typically not detectable on mammograms and is often an unexpected finding during biopsies. It usually presents as a non-palpable, inconspicuous lesion. However, in rare instances, it may be associated with microcalcifications in mammograms and occasionally as a mass or focal area of non-mass enhancement on MRI [36] (Figure 5). Classical LN appears as a low- to intermediate-grade, uniform, intralobular epithelial proliferation of non-cohesive cells, often with prominent intracytoplasmic lumina. A B3 lesion is a non-obligate precursor to breast cancer, classified by the WHO into atypical lobular hyperplasia (ALH) and lobular carcinoma in situ (LCIS). Non-classical LN types, like pleomorphic, apocrine, or florid LCIS, are B5a lesions that necessitate different management due to a higher risk of progression [28].

#### 2.5.2. Upgrade Rate

ALH and LCIS are lesions that act as risk factors, increasing the relative risk by 8 to 10 times [37]. The upgrade rates reported following an LN diagnosis from breast biopsy vary significantly, ranging from 0% to 50% [38]. Increased upgrade rates, ranging from 13 to 18%, are noted for LN linked to mass lesions or calcifications [39]. The strongest predictor of an upgrade is a radiological discrepancy, such as when a mass area is identified on imaging, but the histopathologic diagnosis from CNB reveals LN. However, the upgrade rate is generally lower when the diagnosis is made through VAB. Moreover, there is evidence of an upgrade of these lesions when there is coexisting or adjacent DCIS and/or invasive carcinoma; if the target imaging lesion is attributed to another histological entity rather than LN, the upgrade rate is notably lower [40]. Recently, a meta-analysis that included 16 studies meeting the analysis criteria reduced the upgrade rate, ranging from 3.1 to 5.8% [41].

#### 2.5.3. Current Indications

Several studies indicate that in the event of an LN diagnosis on CNB with radiological–pathological concordance, surgical excision may not be necessary as the upgrade rates are below 5% [38]. The TBCRC 20 trial documented a 13.9% incidence of cancer development after 10 years without affecting breast cancer-specific survival in women diagnosed with LCIS who did not undergo further surgery [42]. The 3rd International Consensus Conference [11] recommends VAE as the step in cases of LN diagnosis on CNB. It does not advocate additional intervention if the target lesion is removed through VAB. European guidelines [10] advise performing VAB/VAE if an LN diagnosis is obtained via CNB, and surveillance is deemed appropriate if there is a pathological–radiological agreement. Surgical excision is only considered in cases of radiological–pathological discrepancy after CNB/VAB diagnosis. Currently, mastectomies are only recommended for individuals with additional high-risk factors, such as a significant family history or pathogenic gene mutations. While, in the past, most patients with classic LCIS underwent a mastectomy, today, 50–80% of women with an LN diagnosis undergo only surgical excision, and mastectomies are performed in only 10–20% of cases [43].

### 2.6. Phyllodes Tumors (PTs)

#### 2.6.1. Radiology and Pathology

**Figure 6:**
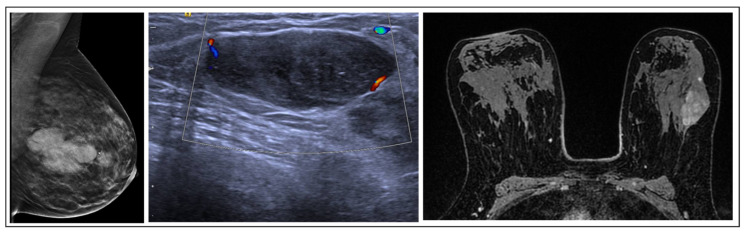
PT radiological features.

PTs generally appear on imaging as round or oval lesions without microcalcifications. On ultrasound, they typically appear as a progressively enlarging fibroadenoma; occasionally, they may display septa indicative of PT, but these are present in only a small percentage of lesions [44]. MRI features of benign phyllodes tumors are similar to fibroadenomas, often with more irregular margins and heterogeneous content. Moreover, MRI has a limited predictive value for distinguishing malignant from benign PT [45] (Figure 6). Histologically, PTs are classified as fibroepithelial lesions composed of epithelial and stromal components, including the more common fibroadenoma. It can be challenging to differentiate fibroadenomas from PTs using CNB due to their similar histological characteristics. Usually, PTs have a more cellular stroma, stromal overgrowth, fragmentation, and mitoses. Marked atypia of stromal cells is rarely seen in cores, and if present, there are usually other features indicating a PT. Based on factors like the margins, stromal cell density, levels of mitotic activity, and proportion of epithelial to stromal factors, the WHO [28] classifies phyllodes tumors (PTs) into benign, which need to be differentiated from fibroadenomas; borderline; and malignant. Malignant PTs are designated as B5 lesions, while both benign and borderline types are labeled as B3.

#### 2.6.2. Upgrade Rate

The upgrade rate to breast cancer following a diagnosis of a PT on biopsy is relatively rare. The existing literature typically addresses how these lesions are managed, often emphasizing the upgrade rate, which refers to the percentage of phyllodes tumors identified in the final histological analysis when biopsy results are inconclusive and indicate that “a phyllodes tumor could not be ruled out.” There is notable variability in these studies; for instance, Rakha et al. [21] discovered that 37% of fibroepithelial lesions identified during biopsy were eventually classified as phyllodes tumors after the final histological evaluation, yet only one 1 of 52 of these lesions was found to be malignant.

#### 2.6.3. Radiology and Pathology

Guidelines suggest excising fibroepithelial lesions larger than 3 cm to rule out PTs. In contrast, others consider the growth rate of the lesion as a more helpful criterion for excision. The distinction between benign fibroadenomas and PTs can be challenging for radiologists and pathologists, and even though biopsies with larger-gauge needles can be considered to achieve this differentiation, surgical excision is the most appropriate solution when a PT cannot be ruled out on biopsy. The 3rd International Consensus Conference [11] recommends excision after a CNB diagnosis of a PT, and if the diagnosis is obtained on VAB, follow-up is justified if the lesion is radiologically removed.

## 3. Discussion

B3 breast lesions include a diverse group of pathological conditions with indeterminate malignant potential. These lesions may be linked to more severe conditions like DCIS, pleomorphic LCIS, or IC. The risk of progression to malignancy differs widely among various types of B3 lesions, with reported upgrade rates ranging from 10% to 35% [5].

Managing B3 lesions poses an important challenge for breast surgeons. As preoperative biopsies become more common in standard practice, there is an escalating necessity for thoughtful decision-making about whether to move forward with surgical removal, VAE, or ongoing surveillance.

**Figure 7:**
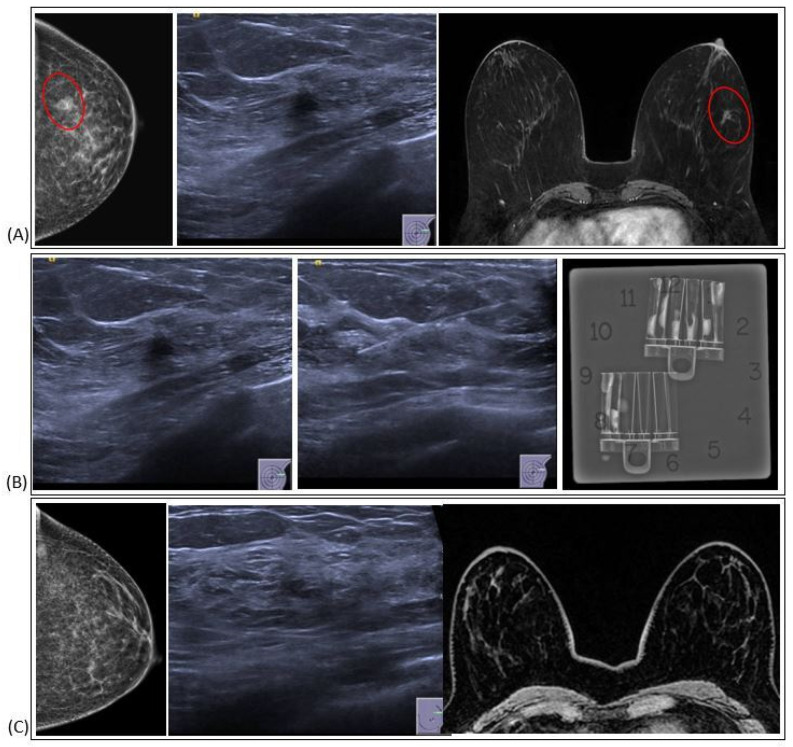
Vacuum-assisted excision (VAE): (**A**) Pre-procedural imaging. The red symbol highlight the position of the target lesion (**B**) VAE (**C**) Radiological assessment after bioptic procedure.

In recent years, there has been a shift towards minimizing surgical interventions, mainly due to the introduction of percutaneous VAE. This less invasive technique allows for the complete removal of smaller B3 lesions, reducing the need for more extensive surgery (Figure 7). According to the 2018 NHS BSP [6], for the management of B3 lesions that are less than 20 mm in size, VAE is the recommended approach. However, in clinical practice, recommendations often still favor surgical excision.

Numerous guidelines have been published concerning treating B3 lesions, each offering slightly different recommendations. The latest European guidelines [10], advocate for a personalized approach based on factors such as atypia, lesion size, biopsy sample size, and patient preferences. Management options may include follow-up, VAE, or surgery, with decisions made within a multidisciplinary context.

The Third Consensus Conference [11] further supports this individualized approach. If a CNB identifies a B3 lesion, the panelists generally advise removal. There is unanimous agreement for lesions with ADH and near-unanimous consensus for those with FEA, LN, PLs with atypia, PTs, and RSs. VAE is a suitable alternative to open excision in routine clinical practice in selected cases.

A summary of the risk factors for upgrading and the current indications for each subtype of B3 lesion is presented in Table 1.

### The Role of Artificial Intelligence (AI) and Genomics in B3 Lesions: Defining Future Perspectives

Incorporating AI and genomic analysis into the study of B3 lesions is essential for advancing our understanding and improving patient outcomes. The integration of these tools is poised to significantly enhance the landscape of diagnosis and treatment, facilitating a shift toward more accurate and individualized medical care. AI has been used in breast screening for decades. Nowadays, its use has broadly expanded to include supporting screening programs, increasing the detection of early-stage BC, and optimizing the cost-effectiveness of second-level procedures [46,47,48]. Barinov et al. [48] assessed that algorithms like Computer-Aided Detection/Computer-Aided Diagnosis and Decision Support Systems can serve as a qualitative integrated tool in the diagnostic pathway, assisting clinicians in their decision-making related to imaging. Moreover, Mango et al. demonstrated that the AI software KOIOS (version 2.0.0.1, Koios Medical) decreased the inter- and intra-observer variability, improving the accuracy in defining the BI-RADS categories [49]. Considering the difficulty in finding an univocal approach toward B3 lesions, AI aims to play a transformative role in their management. AI algorithms, particularly those based on deep learning, can analyze mammogram, ultrasound, and MRI scans to detect and classify suspicious lesions more accurately. AI-based predictive models are being developed to stratify the risk of B3 lesions upgrading to malignancy. These models analyze various features from imaging and biopsy data, and they can help radiologists identify subtle features associated with B3 lesions and distinguish between low- and high-risk ones [46], potentially offering a non-invasive approach or identifying high-risk cases that may require surgical intervention.

Therefore, they can assist clinicians in choosing between conservative management (e.g., surveillance or VAE) and surgical intervention based on risk assessment. These innovations may also lead to more personalized and cost-effective care for patients with B3 lesions. Browne et al. demonstrated that AI algorithms could provide a more reliable evaluation of BI-RADS-3 cases: their data showed that many biopsies with this BI-RADS could have been avoided [50], reducing costs and avoiding women undergoing unnecessary invasive procedures. Studies using algorithms like Computer-Aided Diagnosis (CAD) and Decision Support Systems (DSSs) show that while AI tools have great potential, they are most effective when combined with expert clinical judgment. This is especially important in higher BI-RADS cases, where human input is crucial due to ethical and medical responsibilities [50,51]. It must also be underlined that the data in the literature have some limitations; in fact, all existing studies are retrospective and mainly concern lesions with BI-RADS scores of 4 or 5. Furthermore, there are no studies that concern surgical practice in the case of preoperative B3 diagnosis and underline how AI can influence it. Therefore, new prospective and retrospective data are needed to thoroughly evaluate the integration between AI and surgery in B3 lesions.

Another emerging area of research is the genomic aspect of B3 breast lesions, which focuses on understanding their potential for malignancy. Genomic analysis can support imaging and histopathology to improve the diagnosis, risk assessment, and management of these lesions. Identifying cancer-related expression patterns helps distinguish benign B3 lesions from those with malignant potential. High-risk B3 lesions, for example, may exhibit genetic aberration commonly found in low-grade DCIS (deletion of 16q and gain of 1q) [52]. Moreover, aberrant DNA methylation patterns in B3 lesions could indicate early steps toward malignancy [53].

AI has the potential to revolutionize the management of B3 breast lesions by integrating complex genomic datasets with imaging and clinical data. This comprehensive approach can enhance risk assessment, allowing for the more precise identification of lesions with a higher likelihood of malignancy. By analyzing genomic profiles alongside traditional imaging techniques, clinicians can gain deeper insights into the biological behavior of these lesions. Such integration enables a more nuanced understanding of individual patient risk factors and lesion characteristics, potentially guiding treatment decisions. For example, AI algorithms could identify patterns within genomic data that signal a higher risk, prompting more aggressive management strategies. Additionally, improved risk stratification could optimize the use of resources, directing monitoring or interventions where they are most needed.

Ultimately, leveraging AI’s capabilities enhances the diagnostic accuracy and paves the way for personalized treatment approaches, improving patient outcomes in the challenging landscape of B3 breast lesions. The integration of genomics and AI could transform B3 lesion management by developing non-invasive predictive models, identifying molecular pathways for chemoprevention in high-risk individuals, and offering clinicians real-time decision-making support. Combining genomic insights with AI’s predictive power has great potential to enhance early detection, reduce overtreatment, and personalize care for patients with B3 lesions.

## 4. Conclusions

B3 breast lesions, due to their uncertain malignant potential, represent a complex and evolving challenge in clinical practice. Their heterogeneous nature, combined with the limitations of current diagnostic tools, creates a “grey zone” that complicates the diagnosis and management of these lesions. Although valuable, imaging techniques such as mammography, ultrasound, and MRI often fail to provide definitive answers, and biopsy methods such as CNB and VAB have inherent limitations, including sampling errors and indeterminate results.

Resuming the various indications, surgical intervention is strongly recommended for ADH, but for lesions smaller than 15 mm, VAE may be considered. Surgery is indicated for PLs with atypia; otherwise, PLs without atypia can be safely managed with VABB and with imaging surveillance if entirely excised. For RSs with atypia, open excision or VAE is indicated depending on the lesion size and type of biopsy performed, while for RSs without atypia diagnosed with CNB, VAE is recommended, or, if RSs are diagnosed or completely removed on VABB, only mammographic follow-up is recommended. The upgrade rate for pure FEA is low enough to consider surveillance an effective management option, especially if diagnosed on VAB. Only situations demonstrating pathological–radiological discordance, mass lesions, cases with many residual post-biopsy microcalcifications, or the simultaneous presence of ADH in biopsy specimens should undergo VAE or surgical excision. Isolated LN diagnosed on VAB with adequate radiological–pathological correlation may be managed with follow-up alone. At the same time, surgical excision might be advised for classical LN diagnosed on breast biopsy only if an additional B3 lesion is also detected. In case of a diagnosis obtained via CNB, some guidelines advise VAB/VAE and do not advocate any additional intervention if the diagnosis is confirmed. Surgical excision is usually recommended for PTs diagnosed on biopsy, even if some guidelines consider the growth rate or lesion size over 3 cm as a surgical indication.

Treatment decisions must be carefully tailored to the individual patient, considering factors such as the lesion characteristics, radiological findings, histopathological interpretation, and patient risk factors. This nuanced approach to treatment highlights the need for a multidisciplinary strategy involving radiologists, pathologists, and surgeons to ensure optimal patient care.

This article underlines the importance of further research into improving the diagnostic accuracy and refining treatment algorithms for B3 lesions. Future advances in artificial intelligence, genomics, imaging technology, biopsy techniques, and a deeper understanding of lesion biology will be crucial to enhancing outcomes and minimizing overtreatment or missed diagnoses. Ultimately, a more standardized and evidence-based approach to B3 lesion management will reduce the clinical uncertainty and improve patient care.

## Figures and Tables

**Table 1:** Risk factors for upgrading and current indication for each subtype of B3 lesion.

B3 Subtypes	Upgrade Rate (%)	Risk Factor for Upgrading	Current Indication
Atypical Ductal Hyperplasia (ADH)	5–50%	Radio-pathological mismatch Size > 15 mm Age > 50 years Multifocality in the biopsy specimen Small amount of biopsy tissue	<15 mm VAE can be considered >15 mm surgical excision
Papillary Lesion (PL) no atypia with atypia	9–13.2% 36–47.8%	BI-RADS 4–5 Mass or calcification Peripheral lesion site Palpable lump Size > 1 cm	No atypia -> VAE Atypia -> surgical excision
Radial Scar (RS)	0–40%	Presence of atypia Small amount of biopsy tissue	No atypia -> VAE Atypia -> surgical excision
Flat Epithelial Atypia (FEA)	1–16%	Concurrent proliferative lesion Radio-pathological mismatch Residual calcification after VAB Mass lesion	No ADH/no risk factors -> observation With ADH/risk factors -> VAE vs. surgical excision
Lobular Neoplasia (LN)	0–50%	Mass lesion Radio-pathological mismatch	CNB diagnosis -> VAE VAB diagnosis -> observation Radio-pathological mismatch -> surgical excision
Phyllodes Tumors (PT)	Relatively rare	Growth rate	CNB diagnosis -> surgical excision VAB diagnosis -> observation only if the lesion is entirely removed, otherwise, surgical excision

## Data Availability

No new data were created or analyzed in this study.

## References

[1] American Cancer Society (2024). Cancer Facts and Figures 2024.

[2] Magny S.J., Shikhman R., Keppke A.L. (2024). Breast Imaging Reporting and Data System. StatPearls [Internet].

[3] Ellis I.O., Humphreys S., Michell M., Pinder S.E., Wells C.A., Zakhour H.D., UK National Coordinating Commmittee for Breast Screening Pathology, European Commission Working Group on Breast Screening Pathology (2004). Best Practice No 179. Guidelines for breast needle core biopsy handling and reporting in breast screening assessment. J. Clin. Pathol..

[4] Perry N., Broeders M., de Wolf C., Törnberg S., Holland R., von Karsa L. (2008). European guidelines for quality assurance in breast cancer screening and diagnosis—Fourth Edition—Summary Document. Ann. Oncol..

[5] Bellini C., Nori Cucchiari J., Di Naro F., De Benedetto D., Bicchierai G., Franconeri A., Renda I., Bianchi S., Susini T. (2023). Breast Lesions of Uncertain Malignant Potential (B3) and the Risk of Breast Cancer Development: A Long-Term Follow-Up Study. Cancers.

[6] Pinder S.E., Shaaban A., Deb R., Desai A., Gandhi A., Lee A.H.S., Pain S., Wilkinson L., Sharma N. (2018). NHS Breast Screening multidisciplinary working group guidelines for the diagnosis and management of breast lesions of uncertain malignant potential on core biopsy (B3 lesions). Clin. Radiol..

[7] Rageth C.J., Rubenov R., Bronz C., Dietrich D., Tausch C., Rodewald A.K., Varga Z. (2019). Atypical ductal hyperplasia and the risk of underestimation: Tissue sampling method, multifocality, and associated calcification significantly influence the diagnostic upgrade rate based on subsequent surgical specimens. Breast Cancer.

[8] Catanzariti F., Avendano D., Cicero G., Garza-Montemayor M., Sofia C., Venanzi Rullo E., Ascenti G., Pinker-Domenig K., Marino M.A. (2021). High-risk lesions of the breast: Concurrent diagnostic tools and management recommendations. Insights Imaging.

[9] Yu Y.H., Liang C., Yuan X.Z. (2010). Diagnostic value of vacuum-assisted breast biopsy for breast carcinoma: A meta-analysis and systematic review. Breast Cancer Res. Treat..

[10] Rubio I.T., Wyld L., Marotti L., Athanasiou A., Regitnig P., Catanuto G., Schoones J.W., Zambon M., Camps J., Santini D. (2024). European guidelines for the diagnosis, treatment and follow-up of breast lesions with uncertain malignant potential (B3 lesions) developed jointly by EUSOMA, EUSOBI, ESP (BWG) and ESSO. Eur. J. Surg. Oncol..

[11] Elfgen C., Leo C., Kubik-Huch R.A., Muenst S., Schmidt N., Quinn C., McNally S., van Diest P.J., Mann R.M., Bago-Horvath Z. (2023). Third International Consensus Conference on lesions of uncertain malignant potential in the breast (B3 lesions). Virchows Arch..

[12] Eiada R., Chong J., Kulkarni S., Goldberg F., Muradali D. (2012). Papillary lesions of the breast: MRI, ultrasound, and mammographic appearances. AJR Am. J. Roentgenol..

[13] Kurz K.D., Roy S., Saleh A., Diallo-Danebrock R., Skaane P. (2011). MRI features of intraductal papilloma of the breast: Sheep in wolf’s clothing?. Acta Radiol..

[14] Rakha E.A., Lee A.H., Jenkins J.A., Murphy A.E., Hamilton L.J., Ellis I.O. (2011). Characterization and outcome of breast needle core biopsy diagnoses of lesions of uncertain malignant potential (B3) in abnormalities detected by mammographic screening. Int. J. Cancer.

[15] Nakhlis F., Baker G.M., Pilewskie M., Gelman R., Calvillo K.Z., Ludwig K., McAuliffe P.F., Willey S., Rosenberger L.H., Parker C. (2021). The Incidence of Adjacent Synchronous Invasive Carcinoma and/or Ductal Carcinoma In Situ in Patients with Intraductal Papilloma without Atypia on Core Biopsy: Results from a Prospective Multi-Institutional Registry (TBCRC 034). Ann. Surg. Oncol..

[16] Ni Y., Tse G.M. (2022). Papillary lesions of the breast—Review and practical issues. Semin. Diagn. Pathol..

[17] Zhang X., Liu W., Hai T., Li F. (2021). Upgrade Rate and Predictive Factors for Breast Benign Intraductal Papilloma Diagnosed at Biopsy: A Meta-Analysis. Ann. Surg. Oncol..

[18] Kulka J., Madaras L., Floris G., Lax S.F. (2022). Papillary lesions of the breast. Virchows Arch..

[19] Yan P., DeMello L., Baird G.L., Lourenco A.P. (2021). Malignancy Upgrade Rates of Radial Sclerosing Lesions at Breast Cancer Screening. Radiol. Imaging Cancer.

[20] Choudhery S., Johnson M.P., Larson N.B., Anderson T. (2021). Malignant Outcomes of Architectural Distortion on Tomosynthesis: A Systematic Review and Meta-Analysis. AJR Am. J. Roentgenol..

[21] Rakha E., Beca F., D’Andrea M., Abbas A., Petrou-Nunn W., Shaaban A.M., Kandiyil A., Smith S., Menon S., Elsheikh S. (2019). Outcome of radial scar/complex sclerosing lesion associated with epithelial proliferations with atypia diagnosed on breast core biopsy: Results from a multicentric UK-based study. J. Clin. Pathol..

[22] Lo Gullo R., Vincenti K., Rossi Saccarelli C., Gibbs P., Fox M.J., Daimiel I., Martinez D.F., Jochelson M.S., Morris E.A., Reiner J.S. (2021). Diagnostic value of radiomics and machine learning with dynamic contrast-enhanced magnetic resonance imaging for patients with atypical ductal hyperplasia in predicting malignant upgrade. Breast Cancer Res. Treat..

[23] Farshid G., Buckley E. (2019). Meta-analysis of upgrade rates in 3163 radial scars excised after needle core biopsy diagnosis. Breast Cancer Res. Treat..

[24] Brenner R.J., Jackman R.J., Parker S.H., Evans W.P., Philpotts L., Deutch B.M., Lechner M.C., Lehrer D., Sylvan P., Hunt R. (2002). Percutaneous core needle biopsy of radial scars of the breast: When is excision necessary?. AJR Am. J. Roentgenol..

[25] Quinn E.M., Dunne E., Flanagan F., Mahon S., Stokes M., Barry M.J., Kell M., Walsh S.M. (2020). Radial scars and complex sclerosing lesions on core needle biopsy of the breast: Upgrade rates and long-term outcomes. Breast Cancer Res. Treat..

[26] Ferre R., Kuzmiak C.M. (2022). Upgrade rate of percutaneously diagnosed pure flat epithelial atypia: Systematic review and meta-analysis of 1,924 lesions. J. Osteopath. Med..

[27] Solorzano S., Mesurolle B., Omeroglu A., El Khoury M., Kao E., Aldis A., Meterissian S. (2011). Flat epithelial atypia of the breast: Pathological-radiological correlation. AJR Am. J. Roentgenol..

[28] Tan P.H., Ellis I., Allison K., Brogi E., Fox S.B., Lakhani S., Lazar A.J., Morris E.A., Sahin A., Salgado R. (2020). The 2019 World Health Organization classification of tumours of the breast. Histopathology.

[29] Wahab R.A., Lee S.J., Mulligan M.E., Zhang B., Mahoney M.C. (2021). Upgrade Rate of Pure Flat Epithelial Atypia Diagnosed at Core Needle Biopsy: A Systematic Review and Meta-Analysis. Radiol. Imaging Cancer.

[30] Verschuur-Maes A.H., van Deurzen C.H., Monninkhof E.M., van Diest P.J. (2012). Columnar cell lesions on breast needle biopsies: Is surgical excision necessary? A systematic review. Ann. Surg..

[31] Rudin A.V., Hoskin T.L., Fahy A., Farrell A.M., Nassar A., Ghosh K., Degnim A.C. (2017). Flat Epithelial Atypia on Core Biopsy and Upgrade to Cancer: A Systematic Review and Meta-Analysis. Ann. Surg. Oncol..

[32] Cullinane C., Byrne J., Kelly L., Sullivan M.O., Antony Corrigan M., Paul Redmond H. (2022). The positive predictive value of vacuum assisted biopsy (VAB) in predicting final histological diagnosis for breast lesions of uncertain malignancy (B3 lesions): A systematic review & meta-analysis. Eur. J. Surg. Oncol..

[33] Ditsch N., Wöcke A., Untch M., Jackisch C., Albert U.-S., Banys-Paluchowski M., Bauerfeind I., Blohmer J.-U., Budach W., Dall P. (2022). AGO Recommendations for the Diagnosis and Treatment of Patients with Early Breast Cancer: Update 2022. Breast Care.

[34] Rageth C.J., O’Flynn E.A.M., Pinker K., Kubik-Huch R.A., Mundinger A., Decker T., Tausch C., Dammann F., Baltzer P.A., Fallenberg E.M. (2019). Second International Consensus Conference on lesions of uncertain malignant potential in the breast (B3 lesions). Breast Cancer Res. Treat..

[35] Cha E., Ambinder E.B., Oluyemi E.T., Mullen L.A., Panigrahi B., Rossi J., Di Carlo P.A., Myers K.S. (2022). High-risk lesions in the breast diagnosed by MRI-guided core biopsy: Upgrade rates and features associated with malignancy. Breast Cancer Res. Treat..

[36] Lewin A.A., Mercado C.L. (2020). Atypical Ductal Hyperplasia and Lobular Neoplasia: Update and Easing of Guidelines. AJR Am. J. Roentgenol..

[37] King T.A., Pilewskie M., Muhsen S., Patil S., Mautner S.K., Park A., Oskar S., Guerini-Rocco E., Boafo C., Gooch J.C. (2015). Lobular Carcinoma in Situ: A 29-Year Longitudinal Experience Evaluating Clinicopathologic Features and Breast Cancer Risk. J. Clin. Oncol..

[38] Morrow M., Schnitt S.J., Norton L. (2015). Current management of lesions associated with an increased risk of breast cancer. Nat. Rev. Clin. Oncol..

[39] Hartmann L.C., Degnim A.C., Santen R.J., Dupont W.D., Ghosh K. (2015). Atypical hyperplasia of the breast--risk assessment and management options. N. Engl. J. Med..

[40] Elfgen C., Tausch C., Rodewald A.K., Güth U., Rageth C., Bjelic-Radisic V., Fleisch M., Kurtz C., Gonzalez Diaz J., Varga Z. (2022). Factors Indicating Surgical Excision in Classical Type of Lobular Neoplasia of the Breast. Breast Care.

[41] Shehata M.N., Rahbar H., Flanagan M.R., Kilgore M.R., Lee C.I., Ryser M.D., Lowry K.P. (2020). Risk for Upgrade to Malignancy After Breast Core Needle Biopsy Diagnosis of Lobular Neoplasia: A Systematic Review and Meta-Analysis. J. Am. Coll. Radiol..

[42] Nakhlis F., Gilmore L., Gelman R., Bedrosian I., Ludwig K., Hwang E.S., Willey S., Hudis C., Iglehart J.D., Lawler E. (2016). Incidence of Adjacent Synchronous Invasive Carcinoma and/or Ductal Carcinoma In-situ in Patients with Lobular Neoplasia on Core Biopsy: Results from a Prospective Multi-Institutional Registry (TBCRC 020). Ann. Surg. Oncol..

[43] Brogi E. (2022). The morphologic spectrum of lobular carcinoma in situ (LCIS) observations on clinical significance, management implications and diagnostic pitfalls of classic, florid and pleomorphic LCIS. Virchows Arch..

[44] Evans A.J., Wilson A.R.M., Blamey R.W., Robertson J.F.R., Ellis I.O., Elston C.W. (1998). Phylloides Tumor. Atlas of Breast Disease Management. 50 Illustrative Cases.

[45] Li X., Jiang N., Zhang C., Luo X., Zhong P., Fang J. (2021). Value of conventional magnetic resonance imaging texture analysis in the differential diagnosis of benign and borderline/malignant phyllodes tumors of the breast. Cancer Imaging.

[46] Mendelson E.B. (2019). Artificial Intelligence in Breast Imaging: Potentials and Limitations. AJR Am. J. Roentgenol..

[47] Warren Burhenne L.J., Wood S.A., D’Orsi C.J., Feig S.A., Kopans D.B., O’Shaughnessy K.F., Sickles E.A., Tabar L., Vyborny C.J., Castellino R.A. (2000). Potential contribution of computer-aided detection to the sensitivity of screening mammography. Radiology.

[48] Barinov L., Jairaj A., Paster L., Hulbert W., Mammone R., Podilchuk C. Decision quality support in diagnostic breast ultrasound through artificial intelligence. Proceedings of the IEEE Signal Processing in Medicine and Biology Symposium (SPMB).

[49] Mango V.L., Sun M., Wynn R.T., Ha R. (2020). Should We Ignore, Follow, or Biopsy? Impact of Artificial Intelligence Decision Support on Breast Ultrasound Lesion Assessment. AJR Am. J. Roentgenol..

[50] Browne J.L., Pascual M.Á., Perez J., Salazar S., Valero B., Rodriguez I., Cassina D., Alcázar J.L., Guerriero S., Graupera B. (2023). AI: Can It Make a Difference to the Predictive Value of Ultrasound Breast Biopsy?. Diagnostics.

[51] Barinov L., Jairaj A., Becker M., Seymour S., Lee E., Schram A., Lane E., Goldszal A., Quigley D., Paster L. (2019). Impact of Data Presentation on Physician Performance Utilizing Artificial Intelligence-Based Computer-Aided Diagnosis and Decision Support Systems. J. Digit. Imaging.

[52] Lopez-Garcia M.A., Geyer F.C., Lacroix-Triki M., Marchió C., Reis-Filho J.S. (2010). Breast cancer precursors revisited: Molecular features and progression pathways. Histopathology.

[53] Danforth D.N. (2018). Molecular profile of atypical hyperplasia of the breast. Breast Cancer Res. Treat..

